# Community established best practice recommendations for tephra studies—from collection through analysis

**DOI:** 10.1038/s41597-022-01515-y

**Published:** 2022-07-26

**Authors:** Kristi L. Wallace, Marcus I. Bursik, Stephen Kuehn, Andrei V. Kurbatov, Peter Abbott, Costanza Bonadonna, Katharine Cashman, Siwan M. Davies, Britta Jensen, Christine Lane, Gill Plunkett, Victoria C. Smith, Emma Tomlinson, Thor Thordarsson, J. Douglas Walker

**Affiliations:** 1 US Geological Survey, Alaska Volcano Observatory, Anchorage, USA; 2 SUNY Buffalo, Buffalo, NY USA; 3 Concord University, Athens, WV USA; 4 University of Maine, Orono, ME USA; 5 University of Bern, Bern, Switzerland; 6 University of Geneva, Geneva, Switzerland; 7 University of Oregon, Eugene, OR USA; 8 Swansea University, Swansea, Wales, UK; 9 University of Alberta, Edmonton, Canada; 10 University of Cambridge, Cambridge, UK; 11 Queen’s University Belfast, Belfast, Northern Ireland UK; 12 Research Laboratory for Archaeology and the History of Art, University of Oxford, Oxford, OX1 3TG UK; 13 Trinity College Dublin, Dublin, Ireland; 14 University of Iceland, Reykjavík, Iceland; 15 University of Kansas, Lawrence, KS USA

**Keywords:** Natural hazards, Climate sciences

## Abstract

Tephra is a unique volcanic product with an unparalleled role in understanding past eruptions, long-term behavior of volcanoes, and the effects of volcanism on climate and the environment. Tephra deposits also provide spatially widespread, high-resolution time-stratigraphic markers across a range of sedimentary settings and thus are used in numerous disciplines (e.g., volcanology, climate science, archaeology). Nonetheless, the study of tephra deposits is challenged by a lack of standardization that inhibits data integration across geographic regions and disciplines. We present comprehensive recommendations for tephra data gathering and reporting that were developed by the tephra science community to guide future investigators and to ensure that sufficient data are gathered for interoperability. Recommendations include standardized field and laboratory data collection, reporting and correlation guidance. These are organized as tabulated lists of key metadata with their definition and purpose. They are system independent and usable for template, tool, and database development. This standardized framework promotes consistent documentation and archiving, fosters interdisciplinary communication, and improves effectiveness of data sharing among diverse communities of researchers.

## Introduction

The term ‘tephra’ here indicates any airborne pyroclast produced by an eruption, regardless of grain size, shape, or composition^1^. Tephra plays a critical role in understanding past eruptions^2–10^, long-term behavior of volcanoes^11^, including fundamental processes from the deep earth to the atmosphere, effects of volcanism on climate^12–14^ and the environment^15^, and in providing time-stratigraphic constraints for both geologic events (e.g., geomagnetic reversals^16^, earthquakes, tsunamis^17^) and human history^18,19^. Refer to Table 1 for definitions of terms used herein. Interdisciplinary in its nature, research involving tephra spans a diverse array of scientific and societal interests (Table 2).

**Table 1:** Definitions of terms used in the present work.

Term	Definition
Correlation	The process of establishing a relationship or connection between two or more tephra samples.
Cryptotephra	Typically, distal or sparse tephra deposits that are invisible to the naked eye and found in bogs, glacial, marine or lacustrine environments.
Data field	A place where you can store data. Commonly used to refer to a column in a database or a field in a data entry form or web form. The field may contain data to be entered as well as data to be displayed.
Data type	A particular kind of data item, as defined by the values it can take, the programming language used, or the operations that can be performed on it.
Framework	A basic structure underlying a system, concept, or text.
Isochron	Time-stratigraphic marker horizon
Macrophysical	Physical objects large enough to be directly and individually observed and measured. Herein referring to components of tephra samples.
Microphysical	Physical objects too small to be directly and individually observed and measured. Herein refers to components of tephra samples that require the use of specialized tools to image sample characteristics.
Metadata	A set of data that describes and gives information about other data.
Physical volcanology	A quantitative branch of the study of volcanoes that deals with eruption prediction and forecasting, and measurement of eruption or ejecta parameters or features.
Tephrochronology	A technique that correlates tephra layers between sequences (including paleoenvironmental and archaeological records) to provide relative chronology. These tephra layers can also provide absolute chronology if the source eruption has been dated.
Workbook	A collection of spreadsheets or tabs (e.g., MS Excel)

**Table 2:** Various disciplines that work with tephra in their research.

Discipline	Subdiscipline	Tephra focs
Volcanology	Physical volcanology	eruption characterization^5,6^ eruption frequency and magnitude^1,11,40^, plume dynamics^20,26,28^, eruption mass flux and plume height^22^, ash cloud distribution and fallout (sedimentation) footprint^2,4^, correlation^4,53,61^
	Petrology, geochemistry (magmatic processes)	petrologic and geochemical history of volcano^9,11^, geobarometry, geothermometry, volatile release^13,14^
	Ashfall Impacts	human and animal health^68–72^, environment^15,85^, horticulture^74,86^ infrastructure^73^, transportation^87^
Tephrochronology		tephra-fall frequency^50^, tephra fallout footprint^53,55^
	Geochronology	isochron^51–53,56^, ^14^C reservoir correction, cross-testing and cross-calibrating multiple geochronometers^88^
Paleoclimatology		isochron, volcanic forcing^12–14^
	Paleolimnology	isochron, impact on ecosystem^1,15^
	Paleoceanography	Isochron^89^
	Paleoecology	isochron, impact on ecosystem
Paleontology and evolution		isochron, impacts
Archaeology, human history, human origins		isochron, impacts^18,19^
Anthropology		isochron, impacts, ecology^90^
Glacial geology		Isochron^91^, impacts
Atmospheric science		effects, impacts on atmospheric chemistry^13^
Tectonophysics (paleoseismology)		Isochron^17,92^
Geomorphology		Isochron^93^
Landslide hazards		Isochron, impacts^94,95^
Basin stratigraphy and analysis		Isochron^53^
Paleofire		Isochron^96,97^
Soil science		isochron
Geological data managers		all data collected by above scientists

Because of tephra’s critical role in numerous fields and the diversity of data types involved in its study, standardization of practices and reporting standards, both within and among disciplines, would greatly facilitate mutual understanding, enable the introduction of new research frontiers, and allow for reusability of results even by practitioners outside of the scholarly domain in which the data were originally gathered and results reported. To highlight the importance of documentation and reporting practices, we offer a few examples of data reuse both within and outside the original domain, and introduction of new research possibilities based on legacy data. In 1971, Walker and Croasdale^2^ published a detailed set of grain-size, thickness, and stratigraphic data that were shown on a geocoded map, for the prehistoric Fogo A deposit, Azores. This provided the first case study of a tephra deposit in which comprehensive, well-documented data collection produced a dataset that could be utilized by the broader scientific community. The comprehensive nature of the Fogo A dataset allowed numerous further uses for volcanological analysis, including dispersal index (a measure of deposit spread)^20^ and characterization of phreatomagmatism^21^, eruption rate^22^, thickness-area calculation (another measure of range)^23–25^ total grain size^26^, error analysis and isopach construction^27,28^, and eruption dynamics^24,26^ over multiple decades following the original work. This example shows that comprehensive, well-documented data collection can facilitate wide reuse that leads to insights (likely) unforeseen by the original investigators; we thus credit Walker and Croasdale^2^ with the earliest use of a form of best practices in data collection and reporting for tephra studies. Saxby *et al*.^29^ used distal cryptotephra found by tephrochronologists and archaeologists to constrain physical volcanological parameters, such as cloud height, from the major, past (unwitnessed) eruption that created the Vedde ash in Iceland. This example shows the use of well-documented data collected for two widely different scientific purposes, in yet a third discipline. Recent studies on the use of well-documented tephra deposit information^30–38^ coupled with visual observation of volcanic plume height, to improve estimation of eruption source parameters (i.e., mass flux of gas and ash from a volcanic vent)^39^ further illustrates the value in collecting comprehensive datasets that follow some consistent guidelines, and that can therefore be reused to advance the understanding of how, when and why tephras are produced.

Future innovation and discovery in disciplines that use tephra may thus see phenomenal growth if standardization is widely implemented to generate datasets that are compatible across disciplines. This will improve interdisciplinary collaboration and the interoperability of regional and global tephra datasets. Current, more fragmented, research practices are typically based on, and limited by, physical volcanological datasets local to a volcano^40–42^, regional datasets stored in disconnected databases and offline files of potentially inconsistent geochemical and lithostratigraphic data^43–49^, and a few sparse datasets of volcanic ash distributions at continental and transcontinental scales^50–56^, which are utilized by a diverse community of researchers from different disciplinary backgrounds. With datasets in their current fragmented and inconsistent state, the tephrochronologist struggles to determine the volcanic sources and distribution of isolated tephra or cryptotephra (non-visible ash^57^) layers, especially those transported thousands of kilometers from potential volcanic sources. Jensen *et al*.^55^, for example, showed that the White River ash was long known as a regional stratigraphic marker in Greenland and northern Europe well before it was correlated to an eruption in Alaska. The process of finding potential matches or correlations for a tephra deposit is daunting and determining a unique match may be impossible with the currently available, disconnected datasets. In such circumstances, the paleoclimatologist who found tephra might be unable to link paleoclimate records, and the archaeologist might be unable to use tephrochronology to date a critical cultural site because of uncertainty over the source of a tephra. Similarly, the volcanologist may be faced with data discovery and integration challenges when characterizing the physical and microphysical processes that took place in a volcanic cloud as it dispersed downwind to continental and global scales.

The global nature of tephra studies and the rapid expansion of cryptotephra studies^58–60^ have made advances increasingly dependent on the ability to identify and correlate tephra deposits across broad regions, but this ability is limited by traditional data gathering practices, lack of standardization and inaccessibility of tephra data. These factors, coupled with our growing knowledge of the potential limitations in characterizing tephra using traditional techniques, indicate that implementation of standard practices in tephra sample collection, characterization, correlation, and data reporting is essential to improving the quality and reusability of regional and global tephra datasets. In recognition of this need, a series of multidisciplinary tephra workshops held between 2014–2019 drew consensus from >100 members of the global tephra community on the need to: (a) identify, develop, and share best practices in tephra data collection, analysis, and reporting across different scientific disciplines, and (b) establish common, accessible mechanisms for tephra data archiving and retrieval.

These tephra-community-wide workshops and subsequent developments built upon past efforts and progress to establish best practice methodologies in tephra studies (e.g.^61–63^), with an interdisciplinary viewpoint, on the entire research process from field collection to laboratory analysis and finally to publication and accessible data archiving. Here we focus on the development of a framework of best practices as the first step towards standardization of protocols for the collection, analysis and reporting of tephra data across and within disciplines. The resulting uniformity will facilitate the development, population, and interoperability of globally usable tephra databases (e.g., Tephrabase^43^; RESET^46^; AntT^45^; GeoDIVA^48^ and TephraKam^49^), and the aggregation and reusability of information from multiple datasets. As an expression of this framework, the community has generated best practice recommendations to: (a) ensure consistency in data acquisition and reporting among tephra scientists, regardless of research focus; (b) provide basic, comprehensible metadata requirements, especially for those who collect tephra as a peripheral part of their research; (c) enable the community to create compatible datasets that can be built upon and readily reused; (d) help train students to identify and collect relevant data using checklists; (e) develop templates for publication of supplementary data files and/or upload into databases; (f) inform software tool and data repository development; (g) help journal editors and reviewers to know which essential information should be included in published works, and (h) aid in data management plans required by most funding agencies (e.g., National Science Foundation - NSF, Natural Environmental Research Council - NERC).

Consistency in tephra sample collection, analysis, and reporting attained by the widespread adoption of these best-practice recommendations should ultimately facilitate global collaborative tephra research. We see standardized protocols as a way to finally link diverse research disciplines and scientists; the next step will be to build supporting data management systems and tools to facilitate efficient data access and reuse. Implementation of these best-practice protocols in tephra datasets, data templates, repositories, and digital tools has already begun, and we highlight some examples below.

## Methods

The recommendations for tephra collection, processing, analysis, correlation, and reporting of tephra data presented here are a result of a careful, multi-year process engaging the global interdisciplinary scientific tephra community. They are one product of a series of best practice development efforts that have incorporated broad community input since 2014, and they build upon numerous additional past efforts. Comprehensive representation and participation of >100 tephra scientists from 17 nations (Supplementary Table) ensures a well-vetted set of best practice recommendations. The recommendations are organized as a series of topical, evolving workbooks (spreadsheets with multiple data tabs), openly shared on the Zenodo platform^64^ and which distill all critical components into a readily usable, well-documented, and system-independent format.

### Building blocks to the best practice recommendations

What began as largely separate efforts of the volcanology, tephrochronology, and Quaternary science communities have, in recent years, become gradually more coordinated. In 2006, the Commission on Tephra Hazard Modelling of the International Association of Volcanology and Chemistry of the Earth’s Interior (IAVCEI) conducted a 32-participant field workshop on techniques for assessing maximum clast size distributions, a key parameter for determining the column height of volcanic plumes and for defining eruptive style. This workshop resulted in a series of recommendations summarized by Bonadonna *et al*.^65^.

From 2009–2011, the International focus group on Tephrochronology and Volcanism (INTAV) of the International Union for Quaternary Research (INQUA) – recently renamed the Commission of Tephrochronology (COT) and now associated with IAVCEI – conducted an assessment of analytical data quality^62^ with participation from laboratories that routinely utilize electron probe microanalysis (EPMA) and scanning electron microscopy with energy-dispersive X-ray spectroscopy (SEM-EDS) to characterize volcanic glass compositions. Thirty-eight scientists provided analytical data from 27 instruments at 24 institutions in 9 nations. Among the results are best practice recommendations for analytical methodology and geochemical data reporting along with reference compositions for four widely circulated and well characterized volcanic glasses^62^. Currently a similar project is underway for trace element analyses.

The Tephra in Quaternary Science (TIQS) research group of the UK-based Quaternary Research Association held a 36-participant workshop in 2011 to discuss implications of the 2010 eruptions of Eyjafjallajökull volcano in Iceland for tephrochronology, volcanology, and Quaternary studies. The meeting report^66^ summarizes several essential research needs identified during the workshop, including better geochemical data comparability, routine reporting of tephra grain size data, better protocols for field data collection, and more complete datasets.

The International Volcanic Health Hazard Network (IVHHN)^67^ has developed a set of scientific protocols for rapid collection and analysis of freshly erupted volcanic ash for the assessment of health hazards and includes important workflows for the timing of various analyses (e.g.^68–71^). We defer to the IVHHN for best practices guidelines for analysis of ash for assessment of health impacts but include sample collection guidance for such analyses in the best practice recommendations presented here.

The Volcanic Ash Impacts Working Group of the Cities on Volcanoes Commission of IAVCEI is an international consortium of multidisciplinary geoscientists focused on understanding and mitigating the impacts of ashfall including on critical infrastructure and agriculture, and clean up methodology and disposal, and thus have developed workflows for analysis of tephra following eruptions^72–74^. This working group formally began in 2008 with the goal of pulling the global community together to share experiences and attempt to standardize protocols for ashfall data collection and analysis, create standard data checklists to assess potential societal impact following eruptions, and create ash impacts loss-damage functions for risk calculations. The best practice guidelines for collection of ashfall presented here are in accordance with the goals of this working group, although we explicitly do not include analysis of ash for assessment of societal impacts (including health, as stated above).

### Community-based, interdisciplinary best practice recommendations

Most directly, the present holistic best practice recommendations are the product of international workshops held in 2014, 2017, and 2019. Workshop documents, including consensus reports and summaries, are archived on VHub^75^ (and links therein), and outcomes are summarized at Zenodo^64^. Workshop participants are listed in Supplementary Table.

The first workshop “Tephra 2014 - Maximizing the potential of tephra for multidisciplinary science” specifically aimed to bring together an international and interdisciplinary group to (a) discuss major developments, best practices, future directions, and needs in tephra studies and (b) enhance interdisciplinary collaboration and data sharing. To achieve these goals, the organizers directly contacted >100 scientists across archaeology, geochronology, geoinformatics, geomorphology, limnology, paleoseismology, paleomagnetism, statistics, tectonics, tephrochronology, volcanology, and Quaternary studies and distributed a general call for participants via ≥40 scientific associations, including those involved in the above mentioned “building blocks” to the best practice recommendations presented here. The 70-participant, 4-day workshop was held 3–7 August 2014 in Portland, Oregon, USA. Two consensus^75^ themes emerged from this workshop calling for (a) standardization of tephra field data collection, geochemical analysis, correlation, and data reporting and (b) development of databases to facilitate information access across communities representing different disciplines. All agreed that standardization was a necessary precursor to widespread database development and data sharing. In addition to the consensus report^75^, presentation videos^75^, and a 2014 American Geophysical Union (AGU) annual meeting report poster^76^, the workshop yielded a set of three checklists (predecessors of the current best practice workbooks) covering sample collection, sample analysis, and tephra correlation. These were shared (and physically distributed) during and after the 2015 AGU meeting^77^ to collect community feedback. Feedback was also solicited directly from all 2014 workshop participants and invitees and via postings on several listservs.

A one-day workshop in 2017 was held in conjunction with the quadrennial IAVCEI Scientific Assembly (http://iavcei2017.org/postA_2.html). This second workshop focused on the two consensus themes identified during the 2014 workshop^75^. The ≥50 participants re-affirmed a strong commitment toward standardization and interdisciplinary data sharing and agreed that these efforts would significantly advance tephra research. The three best practice checklists were updated and expanded to include a fourth: tephra data publication. Continued feedback from the tephra community aided in further developing the four checklists.

In 2019, updated checklists, now considered and referred to as “best-practice recommendations” were presented at the International Union for Quaternary Research (INQUA) Congress^78^, and to an International Focus Group on Tephrochronology and Volcanism (INTAV) group by invitation in Dublin, Ireland. In association with these meetings, a small group of 15 researchers, leaders in the fields of tephra collection and analysis, gathered with a focused task: to (a) discuss, debate, and then distill the essential information — i.e., minimum data, methods, and metadata requirements — that must be routinely reported, and to (b) incorporate existing best-practice material as appropriate. The goal of the 2019 workshop was to bring the best-practice recommendations to a place of publication so they could be more widely used by the tephra community at large. The team worked in small, focused groups cataloguing key metadata for the various aspects of tephra collection through to analysis and reporting of data. As a result, the former suite of four checklists were expanded into six separate workbooks focused on metadata recommendations for tephra: (1) collection, (2) processing and preparation, (3) geochemical analysis, (4) physical analysis, (5) physical microanalysis (microscopic imaging), and (6) correlation. These themed workbooks were iterated over the next 10 months and definitions, examples and key references were added to explain metadata. The workbooks and metadata therein are organized in such a way as to be compatible for database development. The first public versions of the best practice recommendation workbooks were archived at Zenodo^64^ in May 2020 and have been revised with community input three times.

## Results

Based on the framework developed by the global scientific tephra community (see Methods), we constructed recommendations in the form of workbooks of tabular data (spreadsheets) that list the most important data types and affiliated metadata required for tephra collection, processing, analysis, and correlation. These recommendation workbooks are linked by categorical relationships, subdivided into logical areas. Major categories are defined to explain their applicability for novice users. Data fields include definitions (what, why, etc.) that provide a shared vocabulary for database and software tool development. Every workbook includes multiple tabs: (1) introduction, (2) metadata and data types, (3) additional resources (references and links), and (4) lists of pre-defined terms for possible inclusion in digital apps (to help reduce typing errors and conform to standard terminology). All data fields that are considered minimum requirements in tephra studies are labelled with an asterisk (*) and bolded.

Six workbooks comprise the best-practice recommendations for (1) *Tephra Collection*, (2) *Tephra Sample Processing & Preparation* (3) *Tephra Physical Analysis*, (4) *Tephra Physical Microanalysis* (or microscopic imaging), (5) *Tephra Geochemical Analysis*, and (6) *Tephra Correlation*. Each workbook covers a different part of the tephra science process, from collecting and documenting data and samples in the field, to processing and preparing samples for analysis, to multiple types of laboratory analysis, to correlating tephra deposits, and finally to reporting the data, methods, and results. Each workbook has been carefully constructed to ensure collection of at least the most essential data (minimum data and their documentation), and to outline optimal, ideal, or best-practice data collection.

For fieldwork, the recommendations emphasize data collection in different contexts, such as excavations, cores, or on the ground surface immediately following an eruption. Sample processing focuses on general processing and preparation for specific types of analyses (not including health impacts). For geochemical laboratory analysis of glasses, minerals, and whole rock, the recommendations emphasize the importance of appropriate secondary standards — i.e., appropriate quality control reference materials — analyzed during the same instrument sessions as unknowns, as the primary and most important documentation of analytical quality. This reference material data should be required for publications, stored in databases, and linked with the corresponding tephra analysis. Past practices have not consistently included such materials or reported such data, severely limiting the ability for others to assess data quality. Similarly, complete analytical results — e.g., all individual point data for EPMA or Laser Ablation Inductively Coupled Mass Spectrometry (LA-ICP-MS), and the individual values for all analyzed elements — must also be reported routinely. For example, analytical techniques like EPMA and ICP-MS are based on signal counting and largely follow Poisson statistics and replicate analyses on a homogeneous material will follow a probability distribution. At low enough concentrations, a portion of that distribution will naturally begin to fall below the single analysis detection limit but are no less valid as members of the population than those analyses which fall above the limit. Therefore, they must be included in analytical results to avoid biasing the dataset by computing statistics without all available data. For physical and microphysical analysis in the field and laboratory, the recommendations emphasize careful thickness measurement as well as grain componentry, size, shape, and mass measurements, which are all important in calculating eruption source parameters. The correlation recommendations emphasize the importance of a multi-parameter approach that includes evaluating geochemical evidence of correlation against independent data, such as age, stratigraphic association, expected deposit thickness, and visual similarity, to substantiate a correlation.

The recommendation workbooks are intended as a catalog of key data and metadata identified by the global scientific tephra community as most important for generating high quality datasets that maximize transparency, data sharing, and collaboration. Minimum requirements are meant as essential practice recommendations, as we recognize that the lack of such information in legacy datasets will limit their re-use, even though these datasets will remain valuable for certain research purposes. Consequently, those developing tephra databases using these recommendations should consider a flexible policy for uploading incomplete legacy data, as well as allowing flexibility in database design to accommodate such data.

The tephra community specifically avoids recommendations of parameters for analytical instrument settings, focusing instead on the basic metadata associated with the various collection and analysis steps, including key instrument metadata that should be recorded during analysis. We recommend that every lab or individual researcher publish their routine lab-specific methods (e.g., details of EPMA, LA-ICPMS settings, details of the epoxy used, etc.) in publicly available systems (e.g., in databases, web sites, on-line data servers, publications), ideally with a permanent digital object identifier (DOI). Minor diversions from a previously documented and cited method can then be reported with the analytical results; major changes in laboratory methodology should result in a new method description. The recommendations include the most commonly performed tephra analyses, and we defer to the literature for specific or evolving analyses, such as those to assess ashfall impacts to society and economy^68–74^.

### Best practice recommendation workbooks

The best practice recommendation workbooks are computer system-independent and can be converted and used with proprietary and non-proprietary software tools: e.g., Google Sheets, Microsoft Excel, Apple Numbers, or OpenOffice. The granular design allows information to be (a) used as a model for database construction, (b) developed into data templates for upload into databases, (c) used to organize data, and (d) used as the basis for data supplemental tables for publication, or (e) as training guides (checklists).

#### Tephra collection

The “Tephra Collection” workbook aims to aid researchers in data collection in field settings from proximal (near the volcano where tephra deposits are typically coarse grained and thick) to ultradistal (thousands of kilometers from the volcano where deposits either are very thin or consist of cryptotephra, trace accumulations of particles less than a few hundred microns that are not visible to the naked eye *in situ*^46^). It is subdivided into tabs that mimic typical workflows based on sampling methodology: (a) project, (b) station or site, (c) subaerial station, (d) core, (e) core drive (section or run), (f) stratum or stratigraphic interval, (g) tephra sample, and (h) eruption-response sampling.

#### Tephra processing & preparation

The “Tephra Processing & Preparation” workbook aims to aid researchers in documenting sample processing performed in a laboratory setting in preparation for analysis. It is subdivided into tabs for general processing and analysis-specific sample preparation: (a) physical sample processing, (b) geochemical and microanalysis (imaging) sample preparation and (c) core processing. Refer to the following sections on tephra “Physical Analysis,” “Microanalysis,” and “Geochemical Analysis” workbooks for subsequent analysis of processed samples and cores.

Tephra analysis includes measurement of both physical and chemical characteristics which may be conducted in the field and/or laboratory. For this reason, we subdivide the Analysis recommendations into “Tephra Physical Analysis,” “Tephra Physical Microanalysis (imaging),” and “Tephra Geochemical Analysis” tabs.

#### Tephra physical analysis

The “Tephra Physical Analysis” workbook includes: (a) physical characteristics (macro), (b) componentry, (c) particle size distribution, (d) maximum clast measurement, (e) density, (f) core, and (g) cryptotephra. Maximum clast measurements are often performed in the field but are described in this workbook rather than in the “Tephra Collection” workbook.

#### Tephra physical microanalysis

The “Tephra Physical Microanalysis” workbook includes commonly used tools for microanalytical sample imaging: (a) polarizing microscope, (c) electron microscope imaging/element mapping (SEM or EPMA), (c) tomography, and (d) other imaging.

#### Tephra geochemical analysis

The “Tephra Geochemical Analysis” workbook includes commonly used tools for geochemical analysis: (a) X-ray fluorescence (XRF), (b) solution ICP-MS, (c) Electron Probe Microanalysis (EPMA) and Scanning Electron Microanalysis (SEM), (d) Laser Ablation Inductively Coupled Mass Spectrometry (LA-ICP-MS), (e) ion probe/SIMS, and (f) geochronology (e.g., radiocarbon). The examples should also guide users on how to report chemical data collected with other tools or methods not specifically addressed: e.g., Inductively coupled plasma - optical emission spectrometry (ICP-OES) or micro XRF. The “Tephra Geochemical Analysis” and “Tephra Physical Microanalysis” tabs also include recommended laboratory instrument metadata and conditions that should be documented as analytical methods, as their documentation allows end users to better understand, evaluate, and replicate analyses.

#### Tephra correlation

The “Tephra Correlation” workbook is designed to aid researchers in using quantitative techniques to link tephra layers from the source volcano to the ultradistal depositional region (and in between) and include: (a) sample, (b) stratigraphy, (c) physical characteristics, (d) geochemistry, (e) correlation, and (f) a correlation confidence check which is a list of questions to help in evaluating the correlation.

## Discussion

The tephra community best-practice recommendations are aimed at maximizing long-term reusability and facilitating sharing of data across disciplines. They are designed to encourage different research groups working with tephra to use similar (standardized) practices for data collection and processing, and to lower barriers for new investigators to enter the field by providing key publications and working examples that they can consult for topics specific to their tephra-related research area.

No single dataset is expected to incorporate all the best-practice recommendations because of the varied nature of real-world tephra studies (e.g., tephrochronology, impacts, eruption characterization). Depending on the goal of the project, only a subset of the guidelines may apply (e.g., excavations, cores, eruption sampling). Rather than focus on one type of research, this community has developed comprehensive recommendations designed to incorporate most types of tephra studies and analyses and to embrace all tephra-using disciplines and research purposes. Following these recommendations will ensure that researchers from different disciplines collect standardized data with well-documented provenance that can be usable beyond their immediate purpose by the greater tephra community. Dissemination of these guidelines is an essential step in realizing the data standardization goal set by the tephra community. They will help change how we do our science and drive future innovations and discoveries.

Following are examples of how the best-practice recommendations have been put into practice thus far. These include development of data and methods templates, field digital tool development, and implementation into open-source data repositories. Among these are efforts to utilize the recommendations in parallel with their development by persons knowledgeable of the process.

### Implementations of the best-practice recommendations

To increase usability of these recommendations and to aid users in adopting the best practices into their workflow, we include examples of datasets that convert the best-practice worksheets into usable formats for data population (that can be used as templates). We have collaborated with the developers of StraboSpot (https://www.strabospot.org/)^79^, a digital field collection tool; System for Earth Sample Registration (SESAR, https://www.geosamples.org/) and EarthChem (https://www.earthchem.org/), open-access geologic data repositories; and Sparrow, lab management software (https://sparrow-data.org/) to incorporate the best practice metadata fields into their systems, to make it easy for users of tephra-specific data.

Kuehn *et al*.^80,81^ converted the best practice workbooks into datasheets or templates. These include examples of field collection templates, datasets of physical sample characteristics (e.g., particle size, maximum clast measurements etc.) and geochemistry. Hopkins *et al*.^82^ and Leicher *at al*.^83^ recently published their regional tephra datasets in the EarthChem open-source data repository using the tephra template (https://www.earthchem.org/communities/tephra/) developed from the best practice workbooks. We encourage the tephra community to contribute links to worked examples like these and new examples (e.g., methods documentation) that can be referenced on Zenodo^64^ along with the best-practice metadata worksheets to be used as citable references, examples, and templates for tephra researchers. Contact the authors if you have working examples to link to the compilation.

The best-practice recommendations serve as the basis for the inclusion of tephra data collection in StraboSpot (https://www.strabospot.org/). StraboSpot is a free, community-developed, geologic mapping and data-recording digital framework optimized for tablets, which archives data in a cloud-based data storage server that is accessible via a web browser. The addition of tephra-specific fields (taken directly from the best practice workbooks) to a new module, StraboSpot Tephra (https://www.strabospot.org/files/StraboSpotTephraHelp.pdf), enables users to consistently collect and report essential tephra data in the field which is then automatically saved to an online data repository. We find that use of StraboSpot for data collection in the field results in more consistent field data than does the use of traditional user-based methods (i.e., field notebooks) because certain metadata are automatically prompted to be recorded such as date, time, and geocoded spot coordinates. The enforcement of data collection, or at least a reminder, is an important feature of StraboSpot and the best-practice recommendations.

SESAR (https://www.geosamples.org/) is a platform for registering information related to samples in a public database, to improve their discoverability. As such, all samples are assigned International Geo Sample Numbers (IGSN) which are globally unique identifiers, allowing samples to be unambiguously cited and linked to data and publications. EarthChem (https://www.earthchem.org/) is an open-access repository that offers data preservation and access, including long-term archiving and registration of data with Digital Object Identifiers (DOIs). The tephra sample registration and data archiving capabilities now introduced into SESAR and EarthChem (https://www.earthchem.org/communities/tephra/), based on the best-practice recommendations, should help ensure discoverability and reuse of data that are stored in these systems. These will also facilitate the publication process as more publishers are enforcing the submission of data to persistent archives. A new tephra portal on the EarthChem website (https://www.earthchem.org/communities/tephra/) allows users to follow simple workflows to register tephra samples at SESAR and submit microanalytical data and methods documentation to EarthChem using templates developed from the best-practice workbooks.

A further step, now under development, is the creation of tephra lab and EPMA plug-ins for the Sparrow laboratory data management system (https://sparrow-data.org/). Sparrow is software for managing analytical data and tracking project- and sample-level metadata. Much like the direct digital data capture in StraboSpot (https://www.strabospot.org/), these plug-ins will enable the capture of sample processing workflows and laboratory data collection while that work is being conducted. This digital-first approach will aid researchers in utilizing best practices and will streamline the process of later submitting information to open repositories, thereby reducing the data archival workload for researchers.

Usage of tephra-specific data templates and tools and the uploading of data to open-source data repositories supports data management for researchers and facilitates faster access to key research by secondary users while meeting FAIR^84^ data principles—findable, accessible, interoperable and reusable.

Because these recommendations have been in development since the 2014 tephra workshop^75^ and have been shared iteratively to solicit feedback from tephra research community, there are numerous examples of their current use by tephra scientists and peer reviewers of tephra data. Since 2019, several journal editors and journal peer reviewers (e.g., GChron, Nature Science Data, Alaska Geological & Geophysical Surveys) have solicited the authors for access to the best-practice recommendations for use in reviewing tephra manuscript submissions. The Zenodo site hosting the recommendation workbooks^64^ has received 2,475 visits and nearly 500 downloads of the workbooks between May 2020 and July 2022. We are aware of several recent studies^80–83^ that have explicitly used the best practice-recommendations for publishing tephra data.

Creating best-practice-recommendation metadata workbooks is a first (but not final) major step to meet the data accessibility goals of the communities that use tephra in their research. It is essential to establish and distribute best practice standards now, early in the process of tephra data globalization, and then develop databases and other derivative frameworks accordingly. Anticipated future uses of the best practice framework include: (a) training guides and checklists for researchers and students doing field, laboratory and interpretative work on tephra deposits, (b) formats or templates for supplementary data for publications and upload to databases, (c) templates for journal editors to help ensure public conformity with standards, (d) DOI-citable method description files linked to analytical data in repositories and publications, and (e) aid in data management plans required by most funding agencies (e.g., NSF, NERC).

The global tephra community encourages all researchers who work with tephra to begin using the recommendations when developing checklists or other tools for data collection, processing, and data supplementary files for publication, because the easiest way to distribute, share, and integrate reusable data is through scientific datasets that follow common practices. Using the recommendations as a guide to what are considered important data and metadata is a key step toward generating high-quality, reusable data that is required for future interconnected databases.

## Supplementary information

Supplementary Information

## Data Availability

The best practice recommendation workbooks^64^ are hosted on the Zenodo platform, an open data server for public comment to ensure dynamic and timely updates and include a DOI for referencing. These workbooks are MS Excel.xlsx files that can be converted to.csv format and opened by other software. The workbooks are considered living documents as we encourage active participation in updating these best practice protocols by the tephra community at large and ask that feedback and comments be emailed directly to the lead author following which they will be vetted by the community. The spreadsheets will be versioned periodically with updated recommendations from the community. We encourage the use of the current version of the recommendations in developing field and analytical templates, data supplements for publications, databases, training guides, etc. Tephra data templates developed from the tephra best practice recommendations and intended for sample registration and data upload to SESAR and EarthChem can be downloaded from the EarthChem tephra portal (https://www.earthchem.org/communities/tephra/) and submitted for upload or used independently as data entry templates for personal use.

## Code availability

This study does not use custom code.
