# Supplementary material for: Community established best practice recommendations for tephra studies—from collection through analysis
Source: Sci Data. 2022 Jul 26;9:447. doi: 10.1038/s41597-022-01515-y (PMC9325882; doi:10.1038/s41597-022-01515-y)
Supplement: Supplementary file 1 — Supplementary Information [file 41597_2022_1515_MOESM1_ESM.docx]

**Appendix 1**. List of participants in tephra data standards workshops, 2014, 2017 and 2019.

| **Last name** | **First name** | **Institution** | **Country** |
| --- | --- | --- | --- |
| Abbott | Peter | Swansea University | UK |
| Adams | Kenneth | Desert Research Institute | USA |
| Addison | Jason | U.S. Geological Survey | USA |
| Albert | Paul | University of Swansea | UK |
| Alfano | Fabrizio | Arizona State University | USA |
| Aubry | Thomas | University of British Columbia | Canada |
| Avard | Geoffroy | OVSICORI-UNA | Costa Rica |
| Baker | Victor | University of Arizona | USA |
| Barker | Simon | Victoria University of Wellington | NZ |
| Bertrand | Sebastien | Ghent University | Belgium |
| Biass | Sebastien | University of Hawaii | USA |
| Bonadonna | Constanza | University of Geneva | Switzerland |
| Bonanati | Christina | GEOMAR Helmholz Center | Germany |
| Bornas | Ma.-Antonia | PhiVolcs | Philippines |
| Buckland | Hannah | University of Bristol | UK |
| Burgess | Seth | USGS-Volcano Science Center | USA |
| Burns | Scott | Portland State University | USA |
| Bursik | Marcus | University at Buffalo | USA |
| Cameron | Cheryl | Alaska Division Geol Geophys | USA |
| Campisano | Chris | Arizona State University | USA |
| Cashman | Kathy | University of Bristol | UK |
| Ciravolo | Amber | University of Nevada Las Vegas | USA |
| Cline | Joy | University of Hawaii at Manoa | USA |
| Comida | Pier-Paolo | Institut Nat’l Recherche Scientif | Canada |
| Courtland | Leah | Georgia Tech | USA |
| Cummings | Michael | Portland State University | USA |
| Danisik | Martin | Curtin University | Australia |
| Davies | Lauren | University of Alberta | Canada |
| Davies | Siwan | Swansea University | UK |
| Deligne | Natalia | U.S. Geological Survey | USA |
| Dennen | Robert | University of Texas at Austin | USA |
| DiMaggio | Erin | Occidental College | USA |
| Dunbar | Nelia | New Mexico Tech | USA |
| Fontijn | Karen | Université Libre de Bruxelles | BE |
| Gardner | Jim | University of Texas | USA |
| Garello | Dominique | Arizona State University | USA |
| Gatti | Emma | University of Cambridge | USA |
| Giachetti | Thomas | University of Oregon | USA |
| Godoi-Millan | Maria-Angelica | Universidad de Magallanes | Chile |
| Gudmundsdottir | Esther | University of Iceland | Iceland |
| **Appendix 1, continued**. List of participants in tephra data standards workshops, 2014, 2017 and 2019. | | | |
| Guilbaud | Marie-Noelle | Universidad Nacional Autónoma | Mexico |
| Hayes | Josh | University of Canterbury | NZ |
| Hornby | Adrian | Ludwig-Maximilians University | UK |
| Hulse | Eva | Archaeological Investigations NW | USA |
| Ickert | Ryan | Berkeley Geochronology Center | USA |
| Iddon | Fiona | University of Cambridge | UK |
| Iverson | Nels | New Mexico Inst Min Tech | USA |
| Janebo | Maria-Helena | University of Iceland | Iceland |
| Jensen | Britta | University of Alberta | Canada |
| Jones | Miriam | U.S. Geological Survey | USA |
| Knott | Tom | University of Leicester | UK |
| Kshirsagar | Pooja | Universidad Nacional Autónoma | Mexico |
| Kuehn | Stephen | Concord University | USA |
| Kurbatov | Andrei | University of Maine | USA |
| Kutterolf | Steffen | GEOMAR Helmholz Center | Germany |
| Lachowycz | Stefan | University of Oxford | UK |
| Lane | Christine | University of Cambridge | UK |
| Lehnert | Kerstin | Columbia University | USA |
| Leonard | Graham | GNS Science | NZ |
| Lim | Chungwan | Seoul National University | Korea |
| Lipshultz | Kathleen | University of Oregon | USA |
| Lit | Catherine | National Inst of Geol Sciences | Philippines |
| Liu | Emma | University of Bristol | UK |
| Lorenzo-Merino | Ainhoa | Universidad Nacional Autónoma | Mexico |
| Lowe | David | University of Waikato | NZ |
| Maharrey | Zebulon | University of Alaska Fairbanks | USA |
| Mahony | Sue | University of Bristol | UK |
| McIntosh | William | NM Bureau of Geology and Min Res | USA |
| McLean | Danielle | University of Oxford | UK |
| Moles | Jonathan | The Open University | UK |
| Muller | James | Harvard University | USA |
| Mulliken | Katherine | University of Alaska Fairbanks | USA |
| Myers | Madison | University of Oregon | USA |
| Newcombe | Megan | Lamont-Doherty Earth Obs | USA |
| Newton | Anthony | University of Edinburgh | UK |
| Oladottir | Bergrun | University of Iceland | Iceland |
| Parcheta | Carolyn | Jet Propulsion Laboratory | USA |
| Pearce | Nicholas | Aberystwyth University | UK |
| Phua | Marcus | Nanyang Technological University | Singapore |
| Pitcher | Bradley | Oregon State University | USA |
| Pittari | Adrian | University of Waikato | NZ |
| **Appendix 1, continued**. List of participants in tephra data standards workshops, 2014, 2017 and 2019. | | | |
| Plank | Terry | Columbia University | USA |
| Plunkett | Gill | Queen's University Belfast | UK |
| Pyne-ODonnell | Sean | Queen's University Belfast | UK |
| Rawson | Harriet | University of Oxford | UK |
| Roland | Thomas | University of Exeter | UK |
| Rotella | Melissa | Victoria University of Wellington | NZ |
| Sarna-Wojcicki | Andrei | U.S. Geological Survey (Emeritus) | USA |
| Schmith | Johanne | Nordic Volcanological Center | Iceland |
| Smith | Victoria | University of Oxford | UK |
| Sourisseau | Delphine | Universidad Nacional Autónoma | Mexico |
| Stoner | Joseph | CEOAS Oregon State University | USA |
| Streck | Martin | Portland State University | USA |
| Streeter | Richard | University of St Andrews | UK |
| Suñe-Puchol | Ivan | Centro de Geociencias, UNAM | Mexico |
| Takarada | Shinji | Geological Survey of Japan, AIST | Japan |
| Thomas | Nicole | University of New Mexico and USGS | USA |
| Tomlinson | Emma | Trinity College Dublin | Ireland |
| Thordarson | Thor | University of Iceland | Iceland |
| Todde | Andrea | Massey University | NZ |
| Van-Eaton | Alexa | USGS Cascades Volcano Obs | USA |
| Van-Wagoner | Nancy | Thompson Rivers University | Canada |
| Waitt | Richard | U.S. Geological Survey | USA |
| Walker | Douglas | University of Kansas | USA |
| Wallace | Kristi | U.S. Geological Survey | USA |
| Walowski | Kristina | University of Oregon | USA |
| Westgate | John | University of Toronto | Canada |
| Williams | Daniel | University of Pittsburgh | USA |
| Zimmerman | Susan | Lawrence Livermore National Lab | USA |
| Zuluaga-Mazo | Indira | Colombia Geological Survey | Colombia |
